# Crosstalk between Innate Lymphoid Cells and Other Immune Cells in the Tumor Microenvironment

**DOI:** 10.1155/2016/7803091

**Published:** 2016-10-12

**Authors:** Fabian Flores-Borja, Sheeba Irshad, Peter Gordon, Felix Wong, Ibrahim Sheriff, Andrew Tutt, Tony Ng

**Affiliations:** ^1^Breast Cancer Now Research Unit, Division of Cancer Studies, Guy's Hospital, King's College London School of Medicine, London SE1 9RT, UK; ^2^Richard Dimbleby Department of Cancer Research, Randall Division & Division of Cancer Studies, King's College London, Guy's Medical School Campus, London SE1 1ULK, UK; ^3^Breast Cancer Now Toby Robins Research Centre, Institute of Cancer Research, London SW3 6JB, UK; ^4^UCL Cancer Institute, Paul O'Gorman Building, University College London, London WC1E 6DD, UK

## Abstract

Our knowledge and understanding of the tumor microenvironment (TME) have been recently expanded with the recognition of the important role of innate lymphoid cells (ILC). Three different groups of ILC have been described based on their ability to produce cytokines that mediate the interactions between innate and adaptive immune cells in a variety of immune responses in infection, allergy, and autoimmunity. However, recent evidence from experimental models and clinical studies has demonstrated that ILC contribute to the mechanisms that generate suppressive or tolerant environments that allow tumor regression or progression. Defining the complex network of interactions and crosstalk of ILC with other immune cells and understanding the specific contributions of each type of ILC leading to tumor development will allow the manipulation of their function and will be important to develop new interventions and therapeutic strategies.

## 1. Introduction

Developments in both basic immunology and tumor biology have increased our knowledge of the interactions between the tumor cells and the immune system. Collectively referred to as the tumor microenvironment (TME), cancers are complex tissues that are comprised of malignant cells and a multitude of stromal cells, such as fibroblasts, epithelial cells, and innate and adaptive immune cells. The TME also includes cells that form blood and lymphatic vasculature, as well as specialized mesenchymal cell types that are unique to each tissue microenvironment [1, 2]. Recently, innate lymphoid cells (ILC) have been added to the list of immune cells that may contribute to the TME [3]. Components within the TME have been shown in experimental models and clinical studies to provide either host protection leading to tumor regression or tumor promotion by providing an immunosuppressive milieu (Table 1). This review will focus primarily on current views of the role of ILC on the control or induction of tumor development and their crosstalk with other immune cells. We also comment on different experimental approaches to further investigate ILC function.

## 2. The Innate Lymphoid Cells (ILC) Family

Lacking a B cell or T cell receptor, ILC are derived from a common lymphoid progenitor and possess a wide range of cell surface markers, many of which have only recently been elucidated [4, 5]. It has been suggested that these antigen receptor-lacking cells play a key role in facilitating and coordinating the innate and adaptive immune responses because they are evolutionary precursors of the adaptive immune system [6]. ILC comprise a small population of mononuclear hematopoietic cells that can be found in the circulation and tissues. Recent moves to propose a uniform nomenclature divide ILC into three subgroups based on the production of Th1, Th2, and Th17 cell associated cytokines [6, 7]. This led to an expert consortium recommending dividing ILC into 3 distinct categories (group 1, group 2, and group 3 ILC) based on the expression of transcription factors, phenotypic markers, and effector cytokine production profiles [6].

### 2.1. Group 1 ILC

Group 1 ILC (ILC1) have a wide range of functions, including cytotoxicity, macrophage activation, immunity to viruses and cancer, and chronic inflammation [8]. ILC1 are dependent on the transcription factor T-bet (encoded by the* Tbx21* gene). There are 2 main subgroups of group 1 ILC in human and mouse—natural killer (NK) cells and non-NK ILC1—and their phenotypic markers and effector cytokines are well defined (Tables 2 and 3). NK cells and non-NK ILC1 can be distinguished based on the expression of the transcription factor Eomesodermin (Eomes); while NK cells express it, non-NK ILC1 do not. [9]. Furthermore, NK cells do not express IL-1 receptor (IL-1R) and therefore do not require development of the transacting T cell-specific transcription factor- (GATA-) 3, which is required by all other ILC including the non-NK ILC1 [10]. Further, only NK cells are distinguished by the expression of CD56 and natural cytotoxicity receptors (NCRs), including NCR1 and NCR2 (also known as NKp46 and NKp44, resp.) [11]. ILC1 produce a range of cytokines upon stimulation by IL-12 or IL-18. Amongst the characteristic cytokines of group 1 ILC are interferon gamma (IFN*γ*) and tumor necrosis factor (TNF-*α*), which are both Th1-related cytokines [12].

### 2.2. Group 2 ILC

Group 2 ILC (ILC2) were first identified in experiments with RAG-deficient mice, in which IL-25 or IL-33 stimulation resulted in increased levels of IL-5 and IL-13 [13], the key characteristic markers and cytokines for the ILC2 (Tables 2 and 3). ILC2 are dependent on epithelial cell-derived cytokines to coordinate responses during inflammation and infection [14] and have an important role in the antihelminth response [15] and development of allergy-related inflammation [16, 17]. This group of ILC displays little heterogeneity, with their development and maintenance dependent on the transcription factors GATA-3 and retinoic acid receptor-related orphan receptor-*α* (ROR*α*), and the growth factor independent 1 transcriptional repressor (Gfi-1), respectively (reviewed in [18]). ILC2 are activated by IL-25, IL-33, and the thymic stromal lymphopoietin (TSLP) and play an important role in type 2 inflammation in the lung and intestine due to their ability to affect T cell responses to allergens through Th2-associated cytokines (IL-4, IL-5, and IL-13) [19, 20]. ILC2 have also been found residing in both human and murine skin. These skin ILC2 are “critically dependent” on activation by TSLP, which is key to promoting skin inflammation [21]. It is well known that the TNF superfamily cytokine TL1A (TNFSF15) promotes ILC2 to produce IL-13* ex vivo*. Furthermore, TL1A costimulates the expansion of ILC2 via their highly expressed TNF-receptor superfamily member DR3 (TNFRSF25), independently of the IL-25 or IL-33 stimulation pathways [22]. Studies with DR3^−/−^ mice demonstrated the importance of ILC2 stimulation by TL1A at the mucosal barriers. The lack of this costimulation leads to deregulated ILC2 functions, as these mice developed gut helminth infections and were unable to mount ILC2 responses in the lungs upon induction of an allergic reaction by nasal papain challenge. The disruption of ILC2 stimulation leads to reduced T cell accumulation and response in T cell dependent allergic models, which was suggested to be potentially beneficial for ILC2-related allergies such as allergic asthma [23].

### 2.3. Group 3 ILC

Group 3 ILC are unique in that they not only are involved in immunity against extracellular bacteria and chronic inflammation but also play a key role in intestinal homeostasis [24, 25] and lymphoid tissue development. Indeed, one of the subgroups of ILC3-lymphoid tissue inducer cells (LTi) was first discovered in the developing lymph nodes, where they play a pivotal role in the formation of lymphoid tissue during organogenesis [26].

In addition to LTi, group 3 ILC include NCR^+^ILC3 and NCR^−^ILC3 (Table 2), which depend on GATA-3 and ROR*γ*t expression. Unlike ILC2, which require ROR*γ*t for their development only, ILC3 require ROR*γ*t for both development and function, while GATA-3 regulates NCR^+^ILC3 as well as NK cells and plays a critical role in the production of IL-22 by these cells [6, 27, 28]. NCR^+^ILC3 express the activating NKp46 or NKp44 receptors [11], and there is also a differential expression of chemokine receptors, whereby only LTi but not NCR^+^ILC3 express CCR6. Upon IL-23 or IL-1*β* stimulation all ILC3 produce IL-22 (Table 3). IL-22 is highly important for ILC3 functions, and studies have shown that mice deficient in lymphotoxin- (LT-) *α*1*β*2 were unable to produce IL-22 in response to colonic infection [29]. In addition to IL-22, LTi and NCR^−^ILC3 also produce the Th17 associated cytokine IL-17 and granulocyte macrophage colony-stimulating factor (GM-CSF) which contribute to the proinflammatory response [30, 31]. Studies in models of intestinal infection have shown that NCR^−^ILC3 are able to produce IFN-*γ* in addition to IL-22 and IL-17 [25]. Interestingly, it was noted that the ability of ILC3 to produce IFN-*γ* is coupled with the disappearance of ROR*γ*t expression and increased expression of T-bet [32]. Other studies have shown that T-bet expression has the ability to induce a phenotype in ILC3 characterized by high levels of IFN-*γ* but not IL-17 [33]. These studies suggest a degree of plasticity between ILC1 and ILC3, similar to that described between Th1 and Th17 cells (reviewed in [6]). This reported plasticity and ability to modify functional phenotype might be important to explain the different effects (pro- or antitumor) of ILC in different models of cancer as will be discussed next.

## 3. Migration and Tissue Distribution of ILC

ILC display a tissue specific distribution with ILC2 and NCR^−^ILC3 preferentially being distributed in skin, while NCR^+^ILC3 are more prominent in the thymus, tonsils, bone marrow, and gut (reviewed in [7]). The mechanism by which the different types of ILC migrate to different tissues is under the control of a differential expression of integrins and chemokine receptors gradients similar to that described for adaptive T cells [2]. Kim et al. have recently shown that ILC1 and ILC3 migrate from the bone marrow to mesenteric lymph nodes in a process controlled by the expression of their homing receptor CCR7. Once in the lymph nodes, ILC1 and ILC3 undergo a homing receptor program switch and express CCR9 and *α*4*β*7 receptors following stimulation by retinoic acid (RA) produced by dendritic cells (DC). This change in their receptor profile then allows migration to intestinal tissue. ILC2 migration, on the other hand, is developmentally controlled as the expression of gut homing receptors occurs in the bone marrow [34]. Further evidence has shown that expression of CXCR6 enables the definition of subpopulations of ILC3 and dictates the distribution of these cells within gut microenvironments. In a model of intestinal infection, CXCL16 released by DC induces the migration of ILC3 to the villus lamina propria where they respond to IL-23 and produce IL-22, which is essential for the release of antimicrobial peptides and infection control [35]. Recent studies using* in vivo* photoconversion to enable cell tracking have also revealed how ILC move from mucosal and peripheral tissues to local draining lymphoid tissues. Mackley et al. have shown that mouse ROR*γ*t^+^ILC migrate from the intestine to draining mesenteric lymph nodes under the influence of chemokine receptor CCR7 [36]. In this way, ILC are enriched in locations such as the marginal sinus, bridging channels, and interfollicular areas where they can interact with trafficking lymphocytes as they recirculate through the blood and lymphatic vessels [37, 38]. Given their anatomical location and ability to rapidly secrete immunoregulatory cytokines and crosstalk with other innate and adaptive immune cells, ILC are proving to be crucial in the regulation of immune responses. ILC respond to environmental stress in immune disorders, infections, allergy, and autoimmunity by producing cytokines that target stromal and epithelial cells, which then mediate the communication between ILC and other immune cells [39]. Understanding the mechanisms and molecules that inhibit [40, 41] or enhance ILC function is vital for the development of immunotherapy for a number of inflammatory diseases, including cancers [42, 43]. In the next sections we will discuss evidence describing the role of ILC in tumor biology.

## 4. Effector Mechanisms of ILC in Cancer

### 4.1. ILC1

**Figure 1:**
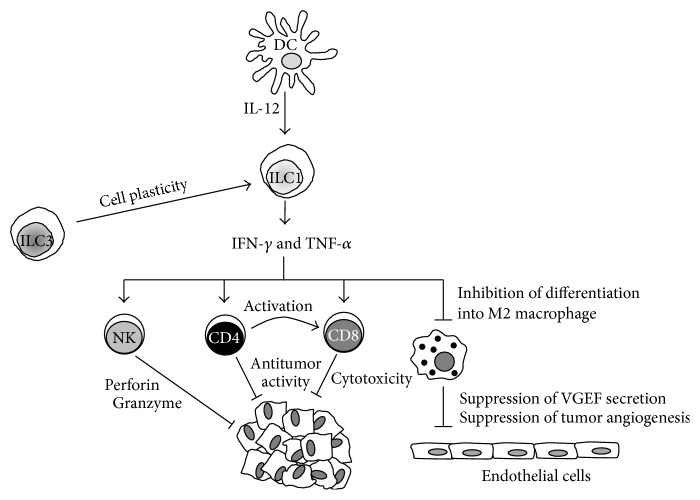
Antitumor activities of group 1 innate lymphoid cells (ILC1). Upon tumor development dendritic cells (DC) are activated and secrete IL-12, which activates ILC1. ILC1 respond to stimulation secreting IFN-*γ* and TNF-*α*, which target and activate different cell types within the tumor microenvironment that display antitumor activities. These cell types include NK cells that kill tumor cells through mechanisms involving perforin and granzyme secretion. CD4^+^ T cells provide costimulation (through cell to cell interactions and secretion of soluble factors) and priming of CD8^+^ cytotoxic T cells which display antitumor cytotoxic activities. IFN-*γ* secreted by ILC1 inhibits the differentiation of tumor-infiltrating macrophages into M2 macrophages providing a mechanism that prevents secretion of vascular endothelial growth factor (VGEF) and tumor angiogenesis. Due to cell plasticity ILC3 can differentiate into ILC within the tumor microenvironment and contribute to the anti- and protumor responses.

The role of NK cells in cancer has been extensively discussed in recent reviews [44] and here we will only focus on non-NK ILC1. New insights from clinical and* in vivo* studies have shown that non-NK ILC1, together with adaptive immune cells, might be involved in responses that either mediate the elimination of tumors (natural cytotoxicity, antibody-dependent cytotoxicity, and phagocytosis) or promote tumor growth and metastasis (Figure 1) [15, 45–47]. Recent reports have started to unravel the role of non-NK ILC1 in tumorigenesis and although they lack expression of granzyme and perforin, they may have similar functions to NK cells in the antitumor response as they share a similar cytokine secretion signature. Recent studies in models of inflammatory bowel disease [32, 48] and intestinal infection with* Toxoplasma gondii* [9] have demonstrated that cells with an ILC1 phenotype secrete IFN-*γ* and TNF-*α* and contribute to the inflammatory response and pathology in response to IL-12 and IL-15. The antiproliferative and proapoptotic properties of IFN-*γ* and TNF-*α*, produced by activated T cells and monocyte-macrophages, are well established in many tumor models. However, whether these cytokines are secreted by ILC and how these might inhibit proliferation and induction of apoptosis need to be addressed. Interestingly, Djenidi et al. have shown that ILC1 had an integrin profile (expression of CD103, integrin alpha E) and a memory-activated phenotype similar to that observed in tumor-infiltrating CD8^+^ T cells, which are tumor-tissue specific and whose presence correlates with improved early stage survival in patients with non-small-cell lung cancer (NSCLC) [49].

### 4.2. ILC2

**Figure 2:**
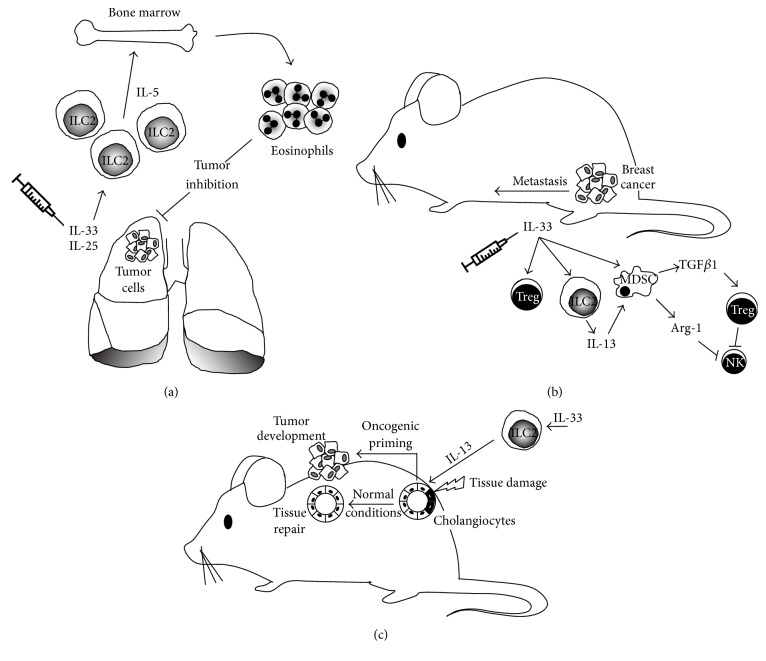
Anti- and protumor activities of group 2 innate lymphoid cells (ILC2). (a) The antitumor activity of ILC2 has been demonstrated in a model of lung metastatic melanoma. IL-33-activated ILC2 produce IL-5, which induces the recruitment and maintenance of eosinophils that display antitumorigenic activity. In contrast, ILC2 can also play an important role in tumor progression as shown in models of liver and breast cancer (b). A study in the 4T1 syngeneic model of breast cancer has shown that IL-33 produced by tumor cells is associated with the induction of a protumor environment characterized by increased numbers of MDSC and Treg. Contributing to the suppressive environment, an increased number of ILC2 secrete IL-5/IL-13 and target MDSC, which in turn secrete TGF*β* (to activate and maintain Treg) and arginase (Arg) to inhibit natural killer (NK) cell activity. Under these immunosuppressive conditions, 4T1 tumors develop and metastasize. (c) Further, evidence of the dual role of ILC2 in tumor development has been highlighted by studies in models of liver cancer. Cell damage of epithelial cells lining the bile ducts (cholangiocytes) in the presence of IL-33-activated ILC2 leads to secretion of IL-13, which under normal conditions is used by epithelial cells to proliferate and induce tissue repair. However, under conditions of oncogenic priming (activation of protumor signaling pathways) the control of epithelial cells proliferation is lost and leads to tumor development.

ILC2 were originally identified in mesenteric lymph nodes and characterized by their ability to prime and stimulate Th2 responses. Following stimulation with IL-25 or IL-33, ILC2 produce IL-5, IL-13 [50, 51], IL-4, IL-6, IL-9, and amphiregulin which induce Th2 differentiation, production of antibodies, and class switching [52]. The effects of ILC2 can be mediated through the secretion of pro-Th2 cytokines or by cell-cell interactions via presentation of MHC class II-associated antigens to T cells or OX40L stimulation [53, 54]. IL-5- and IL-13-producing ILC2 are associated with protective immunity at mucosal surfaces. Clinical studies suggested that increased numbers of ILC2 in peripheral blood, and the cytokines they secrete, could contribute to the immunosuppressive environment maintained by Th2, myeloid-derived suppressor cells (MDSC), and macrophages observed in patients with gastric cancer [55, 56]. Ikutani et al. were amongst the first to show evidence of the role of ILC2 in cancer. Using a mouse model of lung metastatic melanoma, ILC2 were specifically shown to produce IL-5 in response to IL-33 stimulation (Figure 2(a)). Following tumor induction, administration of rIL-33 induced the development of IL-5-producing ILC2, which recruited and maintained eosinophils that induced tumor cell death and prevented tumor metastasis [57], possibly through an IL-4-dependent mechanism [58]. Contrary to an observed antitumor effect and highlighting the dual role of ILC in tumor progression, studies with the 4T1 syngeneic murine model of human triple negative breast cancer have shown that endogenous IL-33, produced by tumor cells, was associated with increased frequencies of TGF*β*-producing MDSC, regulatory T cells (Treg), and ILC2 that expressed IL-5 and IL-13 (Figure 2(b)). The results suggested that ILC2-derived IL-13 targets and activates MDSC to express arginase and nitric oxide synthase, contributing to the establishment of a suppressive environment characterized by increased numbers of Treg and functionally impaired NK cells that allow tumor escape and progression [59]. Preliminary evidence suggesting the involvement of IL-33 and Th2-like cells in other forms of cancer was provided by studies in models of liver disease in which inflammation and fibrosis can result in cancer. The components of IL-33 signaling axis (IL-33/ST2/IL-1RAcP) are increased in human and mouse fibrotic livers but not, interestingly, in human hepatocellular carcinoma [60]. Increased levels of IL-33 were also found in clinical studies in patients with liver cirrhosis and mouse models of hepatic fibrosis. In these studies it was determined that IL-33 secreted by stressed hepatic cells induced the activation and expansion of ILC2. Activated ILC2 responded by secreting IL-13 that targeted stellate cells, which contributed to the mechanisms leading to fibrosis [61, 62]. A recent study highlighting the critical balance that determines whether ILC2 display pro- or anticancer effects has been recently published. In studies by Li et al., they have shown that IL-13 released by IL-33-activated ILC2 promoted proliferation of epithelial cells of the bile ducts or cholangiocytes. Strikingly, cholangiocyte proliferation induced by exogenous IL-33, in a model of biliary injury, promoted epithelial repair (Figure 2(c)). However, the same proliferative effect in mice with oncogenic priming (constitutively active Akt and Hippo pathways) induces cholangiocarcinoma and liver metastasis [62]. These studies suggest that ILC2-activating cytokines might represent potential targets in the design of anticancer therapeutic tools.

### 4.3. ILC3

**Figure 3:**
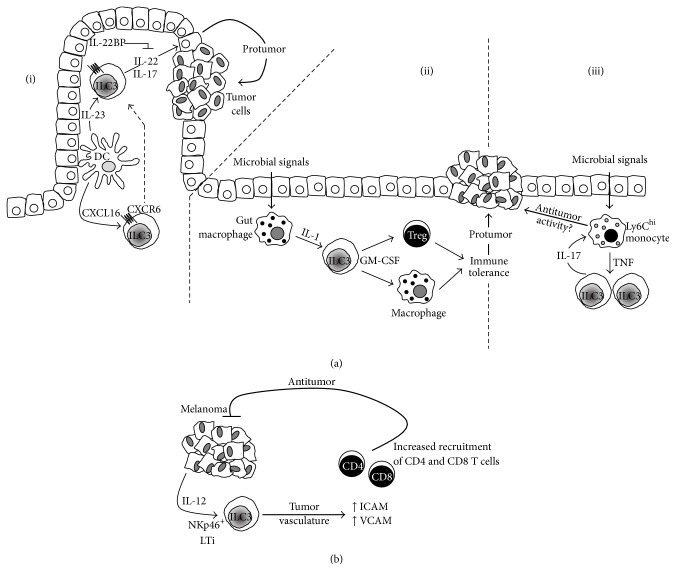
The role of ILC3 in tumor development. Studies in microbe-induced intestinal cancer have provided evidence of the protumor role of ILC3. (a)(i) The expression of CXCR6 allows ILC3 to migrate in response to the CXCL13 gradient and localize in gut microenvironments where they respond to DC-derived IL-23. Upon activation, ILC3 secrete IL-17 and IL-22, which contribute to the inflammatory environment that supports tumor development by inducing proliferation of epithelial cells in a STAT-3-mediated mechanism. The control of IL-22 activity by a soluble receptor, IL-22BP, is important to maintain a fine balance that dictates proliferation and tissue repair or tumor development. Highlighting the importance of crosstalk between ILC and other immune cells, gut macrophages (a)(ii) activated by microbial signals produce the proinflammatory cytokine IL-1, which targets and induces ROR*γ*t^+^ ILC to secrete GM-CSF required to maintain Treg and macrophages. The action of these three cell types creates a tolerant environment that results in tumor progression. Another potential mechanism that might operate in cancer involves Ly6^hi^ monocytes (a)(iii), which produce TNF-*α* following stimulation by microbial signals. The proinflammatory monocytes increase the frequency of IL-17-producing ILC3 and as part of the functional circuit IL-17 acts upon monocytes to increase their microbicidal activity. It would be interesting to evaluate whether this functional circuit results in increased tumoricidal activity by inflammatory monocytes. In subcutaneous melanoma, tumor-derived IL-12 activates NKp46^+^LTi, which induce the tumor microvasculature to express increased levels of ICAM and VCAM. The increased expression of these adhesion molecules allows the infiltration of CD4^+^ and CD8^+^, which mediate tumor suppression. Panel (a)(i) diagram is based on [35].

ILC3 are the group of ILC that have gathered the most evidence suggesting their role in tumor biology. Inflammation due to infection and tissue injury confers an increased risk of cancer and tumorigenesis [63, 64]. In particular, the association between inflammation and colon cancer has been documented for more than 30 years in patients with inflammatory bowel disease (IBD) [65]. IL-17 is an important cytokine linking both innate and adaptive immune responses in infection and autoimmunity [66]. Evidence points to its dual role in both tumorigenesis—inducing angiogenesis [67, 68], tumor evasion, and inhibition of apoptosis—and the control of antitumor responses that activate and recruit NK cells, Treg, and neutrophils to the tumor microenvironment. Clinical and animal models studies have shown correlations between the levels of IL-17 and aggressiveness and tumor progression in gastric, ovarian, breast, and lung carcinomas. The source of IL-17 in those studies was attributed to Th17, mast cells, and tumor-associated macrophages (reviewed in [69]). It is only recently when Kirchberger et al. have demonstrated that microbe-induced colon cancer is directly associated with the accumulation of ILC and IL-17 signaling (Figure 3(a)(i)). Using well-established models of bacteria-induced colon cancer and antibody-based cell depletion and cytokine neutralization protocols, the authors concluded that IL-17 and IL-22 produced by colonic ILC3 contribute to inflammation and tumor development, with an additional role for IL-22 to perpetuate the cancerous state by inducing proliferation of epithelial cells in a STAT-3-mediated mechanism [70]. Whether ILC contribute to recruitment of Treg, MDSC, and protumor M2 macrophages to induce an immunosuppressive protumor environment as shown in mouse models of melanoma and hepatic, cervical, and prostate cancer remains to be fully established [71, 72]. The role of IL-22 in color cancer, however, is complex and not only limited to inducing tumor development, as some studies have shown that IL-22 can display antitumor effects. The control of IL-22 activity is regulated by IL-22 binding protein (IL-22BP), an endogenous neutralizing soluble receptor (Figure 3(a)(i)). Using a model of colon cancer in mice deficient for IL-22BP, Huber et al. demonstrated that IL-22 has an important role in the homeostatic colonic epithelial cell repair. However, in the absence of IL-22BP, the control of the activity of IL-22 by this soluble IL-22 receptor is lost and its protumorigenic activity is triggered [73]. Although, in this study, the source of IL-22 was not attributed to ILC, given the recruitment and presence of these cells in the inflammatory tissue environment, it is likely that they might play a role. In a different model of IBD, the IL-23/IL-17 signaling axis has been reported to operate in the development of mouse gut adenocarcinomas. In the absence of carcinogens or bacterial infection, transgene expression of IL-23 activated IL-23R^+^ILC, which responded by producing IL-17, IFN-*γ*, and IL-22 which contributed to tumor development [74]. These studies suggest that IL-23, IL-17, and IL-22 could be considered as novel therapeutic targets. Interestingly, IL-22-independent crosstalk between ILC3, macrophages, and intestinal microbes has been recently described. Mortha et al. have shown that gut macrophages respond to microbe signals by producing IL-1 (Figure 3(a)(ii)). This proinflammatory cytokine induces ROR*γ*t^+^ILC to secrete GM-CSF, which is required to maintain the tolerance mediated by macrophages, DC, and Treg [75]. This study showed that deficiency of GM-CSF led to reduced numbers of Treg, thus suggesting that targeting the ability of ILC3 to produce GM-CSF could be used as a therapy in cancers where Treg have been described to be increased [76]. Another interesting mechanism of tolerance induction involving ILC3 yet independent of cytokines IL-17, IL22, or IL-23 has been recently described. Hepworth et al. [77] demonstrated that ROR*γ*t^+^ILC3 express MHC-II molecules and present antigens in the absence of costimulatory molecules which allows them to limit microbial-specific CD4^+^ T cell responses which if uncontrolled would cause intestinal inflammation [77]. Whether this is a mechanism that operates in the tumor microenvironment and enables ILC to present tumor antigens and thereby inhibit tumor-specific antigen responses by cytotoxic and effector T cells has not been evaluated. These studies highlight the emerging view on the critical importance of the interactions between resident commensal bacteria and cells of the immune system that maintain gut homeostasis. There are contrasting reports showing, on the one hand, that bacteria are able to promote antitumor responses and, on the other hand, that crosstalk between bacteria and ILC leads to chronic inflammation and cancer development. These observations have clinical importance as many patients with cancer are treated with drugs that might compromise the integrity of intestinal cells, and whether this can have an effect on shifting a balance towards pro- or antitumor responses is something that merits careful consideration [78]. The recently described crosstalk between ILC3 and monocytes highlights another potential mechanism that might operate in cancer. Xiong et al. have shown in a model of lung infection with* Klebsiella pneumoniae* that bacterial infection induces the recruitment of proinflammatory Ly6C^hi^ monocytes, which produce TNF and increase the frequency of IL-17-producing cells (Figure 3(a)(iii)). IL-17 released by ILC3 acts upon recruited monocytes and these cells increase their microbicidal properties and manage to clear off the infecting bacteria [79]. It is very tempting to suggest that such a mechanism with activated monocytes could target tumor cells in a TNF-mediated mechanism. Further to the well-characterized IL-12-dependent cytotoxic activity by Th1 and NK cells, studies with IL-12-deficient mice and mice lacking mature B and T cells (RAG-2 knock-out) have provided clear evidence that IL-12 is an important effector cytokine in the response against melanoma [80, 81]. These results provided a basis to suggest that ILC are involved in the antitumor function of IL-12. Indeed, Eisenring et al. have described an alternative tumor suppression mechanism that depends on the activation of NCR-expressing NKp46^+^LTi, a member of ILC3. Using models of subcutaneous melanoma, IL-12 secreted by tumor cells activated NKp46^+^LTi and these cells induced the tumor microvasculature to express increased levels of ICAM and VCAM. The expression of these two adhesion molecules then allowed the infiltration of CD4^+^ and CD8^+^, which mediate the tumor suppression (Figure 3(b)). Interestingly, IL-12 treatment induced NKp46^+^LTi to secrete IFN-*γ* and LT cytokines, which have well-characterized antitumor and proapoptotic functions. However, the observation that IL-12-mediated tumor rejection operated in IFN-*γ* ^−^, IFN-*γ*R^−^, or perforin-deficient mice suggests that in this model NKp46^+^LTi do not contribute to the antitumor activity of other innate (NK) or adaptive (CD4^+^ and CD8^+^ T) cells in the TME [82]. The role of LTi-like cells has also been highlighted in studies with CCL21-producing melanoma cells. Along with the formation of lymphoid tissue, tumor-derived CCL21 induced the recruitment of LTi-like cells to the TME. Whether the induction of lymphoid-like stroma tissue facilitates immune escape or tolerance has not been fully established [83]. In our own recent studies in patients with triple negative breast cancer (TNBC), we have found evidence suggesting that ROR*γ*t^+^ILC3 might contribute to tumor metastasis through a different mechanism. First, ROR*γ*t^+^ILC3 localize within the primary tumor and an increased number of these cells are associated with tumor migration into lymphatics and subsequent lymph node (LN) metastasis. Using the 4T1.2 syngeneic model of breast cancer we showed that ILC3 recruitment to primary tumors is CCL21-dependent. Once within the tumors, ILC3 stimulate stromal cells to produce CXCL13, which feeds back to promote the production of LT and receptor activator of nuclear factor kappa-B ligand (RANK-L) to promote lymphangiogenesis and enhance tumor cell motility [84]. The identification of ILC3 within the human breast cancer microenvironment is a significant advance for understanding tumor-stromal interactions and their effect on malignant phenotypes in cancer. Analysis of a cohort of 234 breast cancer patient samples showed a correlation of tumors with aggressive invasive properties and a signature of genes expressed by ILC3, enriched for CXCL13, CCL19, CCL21, and the receptors CXCR5 and CCR7 [84]. These findings have potential clinical relevance because they might relate to lymphatic invasion leading to lymph node metastases, a feature routinely assessed by pathologists and considered as poor prognostic factor for breast cancer patients. These results suggest, as seen in other studies, that ILC3 might have different roles in tumor progression depending on the type of tumor and the specific characteristics of the TME. An interesting mechanism through which ILC may influence tumor development is by their role in the formation of tertiary lymphoid structures (TLS). It has been shown that the proinflammatory features of the TME lead to the activation of chemokine signaling pathways and the recruitment of immune cells that contribute to the formation of TLS [85]. TLS are ectopic and highly organized lymphoid formations that develop in inflamed and infected tissues or within or adjacent to primary tumors. These formations present well-defined T and B cell zones, high endothelial venules (HEV), mature DC, germinal centre reactions, and B cell class switch in the B cell follicles, suggesting the generation of adaptive immune responses [86]. These lymphoid aggregates are also characterized by the expression of chemokines (CCL19, CCL21, CXCL10, CXCL12, and CXCL13), adhesion molecules (ICAM-2, ICAM-3, VCAM-1, and MAdCAM-1), and integrins (alphaL, alpha4, and alphaD) [87], which not only attract effector immune cells such as Treg, DC, naïve B cells, and T follicular helper (T_FH_) cells but also determine the architecture and cell segregation in specific compartments. The composition of TLS might be different depending on the tumor type and a growing number of clinical studies suggest that TLS and associated biomarkers correlate with clinical outcome and prognostic value [88]. Studies of patients with melanoma, breast [89], colorectal, lung, pancreas [90], or renal cell carcinomas have shown that the presence of TLS is of positive prognostic value. However, two different studies in patients with breast and renal cell carcinomas have also shown that the presence of TLS has a negative prognostic value (an extensive revision of the clinical studies and trials are reviewed in [86, 91]). Detailed analyses have found that particular cell types within the TLS confer the prognostic value; for instance, CD8^+^ T cells and antibody-producing-plasma cells in ovarian cancer [92] and DC in primary lung tumors [93] confer a positive prognostic value. On the other hand, Joshi et al. have recently shown that tumor-infiltrating Treg in a mouse model of lung adenocarcinoma are increased and found at the tumor margins in tertiary lymphoid structures. At these sites, Treg actively restrain effector T cells [94]. These results, elevated numbers of Treg, correlate with the adverse clinical outcome (poor survival) in patients with breast cancer [95]. In a striking study, Finkin et al. have used a model of hepatocellular carcinoma (HCC) with mice constitutively expressing the active form of IKK-B in hepatocytes to activate the NF-*κ*B pathway. They reported the presence of small clusters of hepatocytes expressing markers of tumor progenitor cells within TLS formed in nontumor sites of the inflamed liver. These clusters progressively coalesced and egressed from the TLS to grow as HCC in all IKK-B-expressing livers. These results suggest that TLS serve as niches supporting tumor cells growth and contribute to recurrence in HCC [96]. Despite the strong correlation between clinical or prognostic value and the presence of TLS in different types of cancer, it is unclear which factors (intrinsic or extrinsic) contribute to their development. Studies from the rheumatology field have suggested a role for ILC in the formation of TLS. Noort et al. have recently shown that TLS within the synovial tissue of some patients with rheumatoid arthritis contain low numbers of CD3^−^RORC^+^ ILC3 which might play a role in TLS formation through a mechanism involving the release of LT*β* and the expansion of follicular DC [97]. Interestingly, in the cancer setting, Carrega et al. have recently reported the presence of NCR^+^ILC3 at the edge of tumor-associated TLS in NSCLC. Increased number of these cells in early stage tumors correlated with the density of intratumoral TLS and predicted favorable clinical outcomes [98]. Further analyses are required to evaluate the role of ILC3 on TLS formation and function in other types of cancer. From the studies mentioned above, it is clear that identifying the mechanisms and cellular components underlying TLS formation will be helpful to understand the pro- and antitumor responses within the tumor microenvironment. This knowledge will be helpful to develop new therapies to promote or inhibit TLS formation. In fact, different drugs and antagonist of the LT*α*/*β*, RANK/RANK-L, and ICOS/ICOS-L signaling pathways are being developed and tested for their potential to manipulate TLS formation (reviewed in [91]). Thus, the paradoxical role of ILC3 in both host protective and tumor-promoting immunosuppressive effects can be associated with the reported functional plasticity that allows these cells to respond to changes in the tumor microenvironment accordingly (reviewed in [6]). Whether ILC display pro- or antitumor activities seems to depend on the type of tumor and stage of development and on the complex network of fine-tuned incoming signals that control cell-cell interactions in the microenvironment. It is likely that the ability to control or influence those interactions will be important for the development of new therapeutic tools to fight cancer. Recent encouraging studies with chemical inhibitors of ROR*γ*t-mediated transcription (such as GSK805) have revealed a transient and specific targeting/inhibition tool for ROR*γ*t^+^ cells. In a recent study of a model of intestinal infection with bacteria, administration of GSK805 reduced cytokine production by Th17, but not ILC3, thus preserving innate immunity. This treatment resulted in a therapeutic response as reduced activity of Th17, but not ILC3, contributed to the control of inflammation in their infection model [99]. Given the nonredundant functions of cytokines produced by T cells and ILC, this cell-specific and transient inhibition of ROR*γ*t^+^ cells might have applications in models of cancer where manipulating and enhancing the antitumor functions of ILC over the proinflammatory functions of Th17 might be beneficial and important to control tumor development. Details on how these cytokines, chemokines, and growth factors allow the cell-cell interactions that maintain the homeostatic balance are currently being explored and will be essential for the development of new therapeutic tools against cancer. In the next section we will discuss imaging approaches to analyze the crosstalk between ILC and other innate and adaptive immune cells.

## 5. Imaging Strategies to Study the Crosstalk and Interactions between ILC and Other Immune Cells

Immunohistochemistry (IHC), immunofluorescence, and flow cytometry methodologies are fundamental tools that have contributed to the identification and our knowledge of the different ILC subsets. In some studies and using IHC techniques, ILC1 have been identified with markers NKp44^+^, CD103^+^, and CD3^−^ [48] and ILC3 using Ror*γ*t^+^IL-7RB^+^CD11c^−^ [100] or CD3^−^CD127^+^CD117^+^ROR*γ*t^+^ [101]. With the use of many different markers and multicolor cytometric analyses, it has been possible to study and determine the presence and changes in ILC populations in normal, disease, and inflammatory settings. However, understanding the roles of ILC in the control of immune responses requires a refined knowledge of the crosstalk and interactions between ILC and other innate and adaptive immune cells. This has been approached through the use of novel imaging techniques using fluorescent probes. Using ILC3 as an example, here we describe some approaches that can be used to examine the role of ILC in the* ex vivo* and* in vivo* settings.

A major complication arising when looking at a subset of cells, such as the ILC, is the necessity to define that subset with a range of different markers. The current series of markers and phenotype accepted for identifying ILC3 require four (five including a nuclear stain) separate fluorophores when imaging tissue to identify the one cell type. If ILC3 are to be imaged along with other cells of interest, the use of serial sections is required, labeling a certain cell type per section and overlaying the images. Alternatively, a multiplex of 6 or 7 fluorescent probes can be used to identify two different cell types on a single slide and imaging on a standard commercial confocal microscope. Using multiple fluorophores in a single slide is generally better accepted as it avoids any slight alterations between sections and the inherent difficulty in obtaining good quality sequentially cut sections. With using such a high number of fluorescent proteins on the same slide, it is common to find spectral bleed-through and cross-excitation between the detected channels. Fluorophore selection including fluorophores with large stokes shift, such as the Brilliant Violet and Pacific Blue/Green/Orange, and the use of quantum dots [102] allow multiplexing on a single excitation laser. This approach, combined with carefully customized microscope configuration, means it is possible to avoid spectral bleed-through altogether [103]. However, in the cases where it is unavoidable, postacquisition correction algorithms can be utilized to correct for unwanted fluorescent emission crosstalk (spectral overlap) by deconvolution/unmixing [104] to remove any remaining overlaps between the fluorophores of the detected channels [105]. Multicolor confocal microscopy provides high-resolution tissue imaging, allowing the labeling of different cell types that require several markers to identify. This provides key localization information in fixed tissue at a particular time point and as such is extremely useful in indicating possible cellular interactions based on the relative cell-cell proximity, which can be further investigated using time-lapse microscopy or other means. A drawback of using confocal microscopy as an approach to imaging ILC3 is the lack of temporal information. Relatively little is known regarding the role these cells play in different scenarios; therefore, the ability to track where these cells migrate and the cells they interact with is fundamental to deciphering their role and function. Multiphoton microscopy (also known as 2-photon microscopy) allows the imaging of fluorescently labeled cells deep within tissue in an* in vivo* setting, visualizing cell behavior in the natural environment. Kinetic information such as cell velocity, track length, meandering index, and displacement can reveal valuable information regarding immune cells activity [106, 107] and the ability to image and track the interactions between different cell subsets is fundamental to further elucidate the roles ILC3 may play in inhibiting particular signaling or receptor pathways. Current commercial multiphoton systems typically have the capacity for 2 to 4 fluorescent probes to be imaged at a time, and using the same multifluorescent labeling as used in confocal microscope is impractical. In this situation, the preferable method is to isolate ILC3 using flow cytometry based cell sorting with the markers for ILC3 (CD3, CD127, CD117, and NKp46), followed by fluorescent labeling of this population using cell tracker dyes [108]. These labeled cells can then be injected intravenously into the target animal and imaged 18–72 hours later, allowing for the cells to migrate to their respective homing tissues [108, 109]. This method allows for the imaging of ILC3 using a single fluorescent channel, leaving the rest for additional labeled cells of interest. As multiphoton microscopy is primarily used for intravital imaging, several approaches could be used to facilitate the imaging of ILC3. The activity of ILC3 within the lymph node could be imaged in a direct manner by surgically exposing the inguinal lymph node [110]. While this approach allows for controllable intravital imaging, it is a terminal procedure and specific experimental time points must be chosen when imaging. This approach could be applied to models of breast cancer with direct exposure and imaging of the draining inguinal lymph node and the primary tumor [111]. Furthermore, in the same tumor model, the use of surgically implanted windows can allow for longitudinal imaging of these organs, allowing repeated imaging of the same tissue throughout tumor development, with or without experimental intervention. A small abdominal window implanted over the inguinal lymph node allows the inguinal lymph node to be reexposed several times for imaging [112]. ILC3 can also be imaged deep within tumors via a mammary window that is inserted in the skin over the lower mammary gland region. Breast cancer cell lines can then be injected/implanted and allowed to grow up into the recess created by the window, therefore allowing a surgery free method of imaging ILC3 behavior over the development of the tumor [111, 113, 114]. The use of this window method can be extended to imaging ILC3 in the lung using a technique developed by Looney et al. [115] which, although terminal, can allow the studying of ILC3 and other target cells in a fully functional lung from chronic obstructive pulmonary disease [116] and lung cancer [98]. Taking advantage of current photo-switchable mouse models provides another tool to examine ILC3 function. In these mice, exposure to ultraviolet light induces changes in fluorescence on the reporter cells [117]. Using this, organs or tissues of interest can be activated using a UV light source, either through surgical exposure or using the aforementioned window systems, and the migration of cells from this tissue was tracked, for instance, as has been shown between intestine and mesenteric lymph nodes [36]. This same approach could be applied to a variety of tumor models to further unveil ILC3 migration properties and the elements that affect them.

## 6. Conclusions

Interest and attention on ILC has increased in recent years and a great deal of information has been gathered. The discovery of this family of innate immune cells, the definition of their phenotypes, and their classification into different subgroups based on their differentiation requirements and biological functions represent important achievements in biomedical research. The description of intricate interactions in different tissues and organs, beyond the gut, with other innate and adaptive immune cells in homeostasis or during different types of infection, inflammatory diseases, and cancer partially highlights the functional importance of these cells. The challenge for future research is to fully understand and decipher the complex and specific contributions of each type of ILC to the control and regulation of immune responses. In particular, further studies on dissecting the detailed nature and implications of the reported plasticity will be required. In the specific case of cancer, observations from the clinic and studies in a variety of animal models have shown opposing abilities of ILC to either promote or repress tumor growth. A better understanding of the precise roles of ILC cell types in tumorigenesis or control will allow understanding of the potential value of manipulation of their functions to develop new interventions and therapeutic strategies.

## Figures and Tables

**Table 1:** Involvement of ILC in different types of tumors. The three different ILC groups have been linked and have been shown to be associated with pro- or antitumor activities in diverse types of tumors. The mechanisms involved include secretion of cytokines and induction of changes in the tumor microenvironment that contribute to control of tumor growth or tumor progression and escape. For details, see main text.

ILC group	Tumor type	Effect	Mechanism
ILC1	Intestinal tumors	Antitumor	Secretion of IFN-*γ*, activation of cytotoxic CD8^+^ T cells, inhibition of macrophage differentiation, and tumor angiogenesis
ILC2	Melanoma	Antitumor	Secretion of IL-5 and recruitment of eosinophils
ILC2	Breast cancer	Protumor	TGF-*β*-mediated induction of MDSC and Treg
ILC2	Cholangiocarcinoma	Protumor	IL-13-mediated proliferation
ILC3	Colon cancer	Protumor	Induction of inflammation by secretion of IL-17 and IL-22
ILC3	Colon cancer	Protumor	IL-22-induced proliferation of tumor cells
ILC3	Melanoma	Antitumor	Increased expression of ICAM and VCAM in tumor vasculature allows CD4^+^ and CD8^+^ infiltration

**Table 2:** Cell markers that define human and mouse ILC.

Marker	ILC1				ILC2		ILC3					
	NK cells		Noncytotoxic ILC1				LTi		NCR^−^ILC3		NCR^+^ILC3	
	H	M	H	M	H	M	H	M	H	M	H	M
CD4^*∗*1^	−	−	−	−	−	−	−	+/−	−	Low	−	−
CD11c	−	−	−	−	−	−	−	−	−	−	−	−
CD25	−/+	−/+	Low	Low	Low	Low	ND	ND	+	+	Low	Low
CD56	+	+	−	−	ND	ND	−	−	−	−	−/+	−/+
CD117	−	−	−	−	+	−/+	High	High	+	+	Low	Low
CD127 (IL-7R*α*)	−/+	−/+	−	−	+	+	+	+	+	+	+	+
NKp44 (NCR2)^*∗*2^	−/+	−	−	−	−	−	−	−	−	−	+	−
ICOS	Low	Low	+	+	+	+	+	+	+	+	+	+
NKp46 (NCR1)	+	+	−	−	−	−	−	−	−	−	+	+
CRTH2 (CD294)	−	−	−	−	+	+	−	−	−	−	−	−
IL-1R	−	−	+	+	+	+	+	+	+	+	+	+
IL-23R	−	−	−	−	ND	ND	+	+	+	+	+	+
IL-12R*β*2	+	+	+	+	−	−	−	−	−	−	−	−
ST2	−	−	−	−	+	+	−	−	−	−	−	−
IL-17RB	−	−	−	−	+	+	−	−	−	−	−	−
NK1.1 (CD161)	−/+	+	−/+	−	+	−	−/+	−	ND	−	+	−/+
Sca1 (Ly6A)^*∗*3^	−	+	−	ND	−	+	−	−	−	+	−	ND
MHC class II	−	−	−	−	−	−	+	+	+	+	−	−
CCR6^*∗*4^	ND	ND	+	−	−/+	−	+	+	+	−	+	−

^*∗*1^There are differences between human and murine CD4 expression. Some murine LTi and a small number of NCR^−^ILC3 express CD4, whereas all human subsets are negative. ^*∗*2^NKp44 is only expressed in human cells. ^*∗*3^Sca1 (also known as Ly6A) is a mouse cell surface protein of the Ly6 family and is not found in human ILC. ^*∗*4^CCR6 expression in human and mouse ILC is different. In mice, CCR6 is not expressed in non-NK ILC1, ILC2, or NCR^+^ILC3. In humans, ILC1, ILC2, and NCR^+^ILC3 all express CCR6. CCR6, C-C chemokine receptor type 6; CRTH2, chemoattractant receptor-homologous molecule expressed on Th2 cells; ICOS, inducible T cell costimulator; IL, interleukin; ILC, innate lymphoid cell; LTi, lymphoid tissue inducer cells; MHC, major histocompatibility complex; NCR, natural cytotoxicity triggering receptor; ND, not determined; NK, natural killer; Sca1, stem cell antigen 1; H, human; M, mouse.

**Table 3:** Effector cytokines produced by ILC.

Cytokines	ILC1		ILC2	ILC3		
	NK cells	Noncytotoxic ILC1		LTi	NCR^−^ILC3	NCR^+^ILC3
IFN*γ*	+	+	−	−	+	−
TNF	+	+	−	−	+	+
Perforin	+	−	−	−	−	−
Granzyme	+	−	−	−	−	−
IL-4	−	−	−/+	−	−	−
IL-5	−	−	+	−	−	−
IL-9	−	−	+	−	−	−
IL-13	−	−	+	−	−	−
IL-17A	−	−	−	+	+	−
IL-22	−	−	−	+	+	+
Areg	−	−	+	−	−	−
LT-*α*1*β*2	−	−	−	+	+	+
GM-CSF	+	−	−	+	+	+

Areg, amphiregulin; GM-CSF, granulocyte macrophage colony-stimulating factor; INF*γ*, interferon gamma; LT, lymphotoxin; and TNF, tumor necrosis factor.

## Additional Points

*Review Search Strategy.* This is a narrative review. We searched PUBMED for original articles focusing on innate lymphoid cells and cancer published in the last 10 years, from 2006 to 2016. Search terms included “Group 1, 2, and 3 Innate lymphoid cells and tumor”, “ILC1, 2, 3 and cancer”, and “Innate lymphoid cells and tumor microenvironment”. All papers identified were English-language, full-text papers. We also searched the reference lists of identified articles for further papers.

## References

[1] Turley S. J., Cremasco V., Astarita J. L. (2015). Immunological hallmarks of stromal cells in the tumour microenvironment. *Nature Reviews Immunology*.

[2] Agace W. W. (2008). T-cell recruitment to the intestinal mucosa. *Trends in Immunology*.

[3] Vesely M. D., Kershaw M. H., Schreiber R. D., Smyth M. J. (2011). Natural innate and adaptive immunity to cancer. *Annual Review of Immunology*.

[4] Lanier L. L. (2013). Shades of grey-the blurring view of innate and adaptive immunity. *Nature Reviews Immunology*.

[5] Eberl G., Di Santo J. P., Vivier E. (2015). The brave new world of innate lymphoid cells. *Nature Immunology*.

[6] Spits H., Artis D., Colonna M. (2013). Innate lymphoid cells—a proposal for uniform nomenclature. *Nature Reviews Immunology*.

[7] Hazenberg M. D., Spits H. (2014). Human innate lymphoid cells. *Blood*.

[8] Biron C. A., Nguyen K. B., Pien G. C., Cousens L. P., Salazar-Mather T. P. (1999). Natural killer cells in antiviral defense: function and regulation by innate cytokines. *Annual Review of Immunology*.

[9] Klose C. S. N., Flach M., Möhle L. (2014). Differentiation of type 1 ILCs from a common progenitor to all helper-like innate lymphoid cell lineages. *Cell*.

[10] Yagi R., Zhong C., Northrup D. L. (2014). The transcription factor GATA3 is critical for the development of all IL-7R*α*-expressing innate lymphoid cells. *Immunity*.

[11] Moretta A., Bottino C., Vitale M. (2001). Activating receptors and coreceptors involved in human natural killer cell-mediated cytolysis. *Annual Review of Immunology*.

[12] Walker J. A., Barlow J. L., McKenzie A. N. J. (2013). Innate lymphoid cells—how did we miss them?. *Nature Reviews Immunology*.

[13] Fort M. M., Cheung J., Yen D. (2001). IL-25 Induces IL-4, IL-5, and IL-13 and Th2-associated pathologies in vivo. *Immunity*.

[14] Huang Y., Guo L., Qiu J. (2015). IL-25-responsive, lineage-negative KLRG1 hi cells are multipotential ‘inflammatory’ type 2 innate lymphoid cells. *Nature Immunology*.

[15] Walker J. A., McKenzie A. N. J. (2013). Development and function of group 2 innate lymphoid cells. *Current Opinion in Immunology*.

[16] Licona-Limón P., Kim L. K., Palm N. W., Flavell R. A. (2013). TH2, allergy and group 2 innate lymphoid cells. *Nature Immunology*.

[17] Chang Y.-J., Kim H. Y., Albacker L. A. (2011). Innate lymphoid cells mediate influenza-induced airway hyper-reactivity independently of adaptive immunity. *Nature Immunology*.

[18] Zhu J. (2015). T helper 2 (Th2) cell differentiation, type 2 innate lymphoid cell (ILC2) development and regulation of interleukin-4 (IL-4) and IL-13 production. *Cytokine*.

[19] Zaiss D. M., Yang L., Shah P. R., Kobie J. J., Urban J. F., Mosmann T. R. (2006). Amphiregulin, a TH2 cytokine enhancing resistance to nematodes. *Science*.

[20] Monticelli L. A., Sonnenberg G. F., Abt M. C. (2011). Innate lymphoid cells promote lung-tissue homeostasis after infection with influenza virus. *Nature Immunology*.

[21] Kim B. S., Siracusa M. C., Saenz S. A. (2013). TSLP elicits IL-33-independent innate lymphoid cell responses to promote skin inflammation. *Science Translational Medicine*.

[22] Yu X., Pappu R., Ramirez-Carrozzi V. (2014). TNF superfamily member TL1A elicits type 2 innate lymphoid cells at mucosal barriers. *Mucosal Immunology*.

[23] Richard A. C., Tan C., Hawley E. T. (2015). The TNF-family ligand TL1A and its receptor DR3 promote T cell-mediated allergic immunopathology by enhancing differentiation and pathogenicity of IL-9-producing T cells. *The Journal of Immunology*.

[24] Satoh-Takayama N., Vosshenrich C. A. J., Lesjean-Pottier S. (2008). Microbial flora drives interleukin 22 production in intestinal NKp46+ cells that provide innate mucosal immune defense. *Immunity*.

[25] Vonarbourg C., Mortha A., Bui V. L. (2010). Regulated expression of nuclear receptor ROR*γ*t confers distinct functional fates to NK cell receptor-expressing ROR*γ*t^+^ innate lymphocytes. *Immunity*.

[26] Mebius R. E. (2003). Organogenesis of lymphoid tissues. *Nature Reviews Immunology*.

[27] Sun Z., Unutmaz D., Zou Y.-R. (2000). Requirement for ROR*γ* in thymocyte survival and lymphoid organ development. *Science*.

[28] Zhong C., Cui K., Wilhelm C. (2016). Group 3 innate lymphoid cells continuously require the transcription factor GATA-3 after commitment. *Nature Immunology*.

[29] Ota N., Wong K., Valdez P. A. (2011). IL-22 bridges the lymphotoxin pathway with the maintenance of colonic lymphoid structures during infection with Citrobacter rodentium. *Nature Immunology*.

[30] Takatori H., Kanno Y., Watford W. T. (2009). Lymphoid tissue inducer-like cells are an innate source of IL-17 and IL-22. *The Journal of Experimental Medicine*.

[31] El-Behi M., Ciric B., Dai H. (2011). The encephalitogenicity of TH 17 cells is dependent on IL-1- and IL-23-induced production of the cytokine GM-CSF. *Nature Immunology*.

[32] Bernink J. H., Peters C. P., Munneke M. (2013). Human type 1 innate lymphoid cells accumulate in inflamed mucosal tissues. *Nature Immunology*.

[33] Powell N., Walker A. W., Stolarczyk E. (2012). The transcription factor T-bet regulates intestinal inflammation mediated by interleukin-7 receptor+ innate lymphoid cells. *Immunity*.

[34] Kim M. H., Taparowsky E. J., Kim C. H. (2015). Retinoic acid differentially regulates the migration of innate lymphoid cell subsets to the gut. *Immunity*.

[35] Satoh-Takayama N., Serafini N., Verrier T. (2014). The chemokine receptor CXCR6 controls the functional topography of interleukin-22 producing intestinal innate lymphoid cells. *Immunity*.

[36] Mackley E. C., Houston S., Marriott C. L. (2015). CCR7-dependent trafficking of ROR*γ*
^+^ ILCs creates a unique microenvironment within mucosal draining lymph nodes. *Nature Communications*.

[37] Bajénoff M., Glaichenhaus N., Germain R. N. (2008). Fibroblastic reticular cells guide T lymphocyte entry into and migration within the splenic T cell zone. *Journal of Immunology*.

[38] Lane P. J. L., Gaspal F. M., McConnell F. M., Withers D. R., Anderson G. (2012). Lymphoid tissue inducer cells: pivotal cells in the evolution of CD4 immunity and tolerance?. *Frontiers in Immunology*.

[39] Magri G., Miyajima M., Bascones S. (2014). Innate lymphoid cells integrate stromal and immunological signals to enhance antibody production by splenic marginal zone B cells. *Nature Immunology*.

[40] Huh J. R., Leung M. W., Huang P. (2011). Digoxin and its derivatives suppress TH17 cell differentiation by antagonizing RORgammat activity. *Nature*.

[41] Solt L. A., Kumar N., Nuhant P. (2011). Suppression of TH17 differentiation and autoimmunity by a synthetic ROR ligand. *Nature*.

[42] De Visser K. E., Eichten A., Coussens L. M. (2006). Paradoxical roles of the immune system during cancer development. *Nature Reviews Cancer*.

[43] Sonnenberg G. F., Artis D. (2015). Innate lymphoid cells in the initiation, regulation and resolution of inflammation. *Nature Medicine*.

[44] Carotta S. (2016). Targeting NK cells for anticancer immunotherapy: clinical and preclinical approaches. *Frontiers in Immunology*.

[45] Grivennikov S. I., Greten F. R., Karin M. (2010). Immunity, inflammation, and cancer. *Cell*.

[46] Nowarski R., Gagliani N., Huber S., Flavell R. A. (2013). Innate immune cells in inflammation and cancer. *Cancer Immunology Research*.

[47] Vallentin B., Barlogis V., Piperoglou C. (2015). Innate lymphoid cells in cancer. *Cancer Immunology Research*.

[48] Fuchs A., Vermi W., Lee J. S. (2013). Intraepithelial type 1 innate lymphoid cells are a unique subset of IL-12- and IL-15-responsive IFN-*γ*-producing cells. *Immunity*.

[49] Djenidi F., Adam J., Goubar A. (2015). CD8^+^CD103^+^ tumor-infiltrating lymphocytes are tumor-specific tissue-resident memory T cells and a prognostic factor for survival in lung cancer patients. *The Journal of Immunology*.

[50] Fallon P. G., Ballantyne S. J., Mangan N. E. (2006). Identification of an interleukin (IL)-25-dependent cell population that provides IL-4, IL-5, and IL-13 at the onset of helminth expulsion. *Journal of Experimental Medicine*.

[51] Moro K., Yamada T., Tanabe M. (2010). Innate production of T(H)2 cytokines by adipose tissue-associated c-Kit(+)Sca-1(+) lymphoid cells. *Nature*.

[52] Dancescu M., Rubio-Trujillo M., Biron G., Bron D., Delespesse G., Sarfati M. (1992). Interleukin 4 protects chronic lymphocytic leukemic B cells from death by apoptosis and upregulates Bcl-2 expression. *The Journal of Experimental Medicine*.

[53] Oliphant C. J., Hwang Y. Y., Walker J. A. (2014). MHCII-mediated dialog between group 2 innate lymphoid cells and CD4+ T cells potentiates type 2 immunity and promotes parasitic helminth expulsion. *Immunity*.

[54] Drake L. Y., Iijima K., Kita H. (2014). Group 2 innate lymphoid cells and CD4^+^ T cells cooperate to mediate type 2 immune response in mice. *Allergy*.

[55] Yang Z., Zhang B., Li D. (2010). Mast cells mobilize myeloid-derived suppressor cells and Treg cells in tumor microenvironment via IL-17 pathway in murine hepatocarcinoma model. *PLoS ONE*.

[56] Bie Q., Zhang P., Su Z. (2014). Polarization of ILC2s in peripheral blood might contribute to immunosuppressive microenvironment in patients with gastric cancer. *Journal of Immunology Research*.

[57] Ikutani M., Yanagibashi T., Ogasawara M. (2012). Identification of innate IL-5-producing cells and their role in lung eosinophil regulation and antitumor immunity. *The Journal of Immunology*.

[58] Tepper R. I., Coffman R. L., Leder P. (1992). An eosinophil-dependent mechanism for the antitumor effect of interleukin-4. *Science*.

[59] Jovanovic I. P., Pejnovic N. N., Radosavljevic G. D. (2014). Interleukin-33/ST2 axis promotes breast cancer growth and metastases by facilitating intratumoral accumulation of immunosuppressive and innate lymphoid cells. *International Journal of Cancer*.

[60] Marvie P., Lisbonne M., L'Helgoualc'h A. (2010). Interleukin-33 overexpression is associated with liver fibrosis in mice and humans. *Journal of Cellular and Molecular Medicine*.

[61] Mchedlidze T., Waldner M., Zopf S. (2013). Interleukin-33-dependent innate lymphoid cells mediate hepatic fibrosis. *Immunity*.

[62] Li J., Razumilava N., Gores G. J. (2014). Biliary repair and carcinogenesis are mediated by IL-33-dependent cholangiocyte proliferation. *The Journal of Clinical Investigation*.

[63] Elinav E., Nowarski R., Thaiss C. A., Hu B., Jin C., Flavell R. A. (2013). Inflammation-induced cancer: crosstalk between tumours, immune cells and microorganisms. *Nature Reviews Cancer*.

[64] Herszényi L., Barabás L., Miheller P., Tulassay Z. (2015). Colorectal cancer in patients with inflammatory bowel disease: the true impact of the risk. *Digestive Diseases*.

[65] Morson B. C. (1985). Precancer and cancer in inflammatory bowel disease. *Pathology*.

[66] Gaffen S. L., Jain R., Garg A. V., Cua D. J. (2014). The IL-23-IL-17 immune axis: from mechanisms to therapeutic testing. *Nature Reviews Immunology*.

[67] Numasaki M., Fukushi J.-I., Ono M. (2003). Interleukin-17 promotes angiogenesis and tumor growth. *Blood*.

[68] Chen X., Xie Q., Cheng X. (2010). Role of interleukin-17 in lymphangiogenesis in non-small-cell lung cancer: enhanced production of vascular endothelial growth factor C in non-small-cell lung carcinoma cells. *Cancer Science*.

[69] Qian X., Chen H., Wu X., Hu L., Huang Q., Jin Y. (2015). Interleukin-17 acts as double-edged sword in anti-tumor immunity and tumorigenesis. *Cytokine*.

[70] Kirchberger S., Royston D. J., Boulard O. (2013). Innate lymphoid cells sustain colon cancer through production of interleukin-22 in a mouse model. *Journal of Experimental Medicine*.

[71] Li Q., Liu L., Zhang Q., Liu S., Ge D., You Z. (2014). Interleukin-17 indirectly promotes M2 macrophage differentiation through stimulation of COX-2/PGE2 pathway in the cancer cells. *Cancer Research and Treatment*.

[72] He D., Li H., Yusuf N. (2010). IL-17 promotes tumor development through the induction of tumor promoting microenvironments at tumor sites and myeloid-derived suppressor cells. *The Journal of Immunology*.

[73] Huber S., Gagliani N., Zenewicz L. A. (2012). IL-22BP is regulated by the inflammasome and modulates tumorigenesis in the intestine. *Nature*.

[74] Chan I. H., Jain R., Tessmer M. S. (2014). Interleukin-23 is sufficient to induce rapid de novo gut tumorigenesis, independent of carcinogens, through activation of innate lymphoid cells. *Mucosal Immunology*.

[75] Mortha A., Chudnovskiy A., Hashimoto D. (2014). Microbiota-dependent crosstalk between macrophages and ILC3 promotes intestinal homeostasis. *Science*.

[76] Banerjee A., Vasanthakumar A., Grigoriadis G. (2013). Modulating T regulatory cells in cancer: how close are we?. *Immunology & Cell Biology*.

[77] Hepworth M. R., Monticelli L. A., Fung T. C. (2013). Innate lymphoid cells regulate CD4+ T-cell responses to intestinal commensal bacteria. *Nature*.

[78] Viaud S., Daillere R., Boneca I. G. (2015). Gut microbiome and anticancer immune response: really hot Sh∗t!. *Cell Death and Differentiation*.

[79] Xiong H., Keith J. W., Samilo D. W., Carter R. A., Leiner I. M., Pamer E. G. (2016). Innate lymphocyte/Ly6C^hi^ monocyte crosstalk promotes *Klebsiella Pneumoniae* clearance. *Cell*.

[80] Carnaud C., Lee D., Donnars O. (1999). Cutting edge: cross-talk between cells of the innate immune system: NKT cells rapidly activate NK cells. *The Journal of Immunology*.

[81] Kodama T., Takeda K., Shimozato O. (1999). Perforin-dependent NK cell cytotoxicity is sufficient for anti-metastatic effect of IL-12. *European Journal of Immunology*.

[82] Eisenring M., vom Berg J., Kristiansen G., Saller E., Becher B. (2010). IL-12 initiates tumor rejection via lymphoid tissue-inducer cells bearing the natural cytotoxicity receptor NKp46. *Nature Immunology*.

[83] Shields J. D., Kourtis I. C., Tomei A. A., Roberts J. M., Swartz M. A. (2010). Induction of lymphoidlike stroma and immune escape by tumors that express the chemokine CCL21. *Science*.

[84] Irshad S., Lawler K., Evans R. (2014). Effect of lymphoid tissue inducer cells on lymphatic tumor cell invasion via activation of the RANKL/RANK axis within triple-negative breast cancers. *Journal of Clinical Oncology*.

[85] Weinstein A. M., Storkus W. J. (2015). Therapeutic lymphoid organogenesis in the tumor microenvironment. *Advances in Cancer Research*.

[86] Dieu-Nosjean M.-C., Goc J., Giraldo N. A., Sautès-Fridman C., Fridman W. H. (2014). Tertiary lymphoid structures in cancer and beyond. *Trends in Immunology*.

[87] de Chaisemartin L., Goc J., Damotte D. (2011). Characterization of chemokines and adhesion molecules associated with T cell presence in tertiary lymphoid structures in human lung cancer. *Cancer Research*.

[88] Fridman W. H., Galon J., Dieu-Nosjean M.-C. (2011). Immune infiltration in human cancer: prognostic significance and disease control. *Current Topics in Microbiology and Immunology*.

[89] Lee H. J., Park I. A., Song I. H. (2016). Tertiary lymphoid structures: prognostic significance and relationship with tumour-infiltrating lymphocytes in triple-negative breast cancer. *Journal of Clinical Pathology*.

[90] Hiraoka N., Ino Y., Yamazaki-Itoh R., Kanai Y., Kosuge T., Shimada K. (2015). Intratumoral tertiary lymphoid organ is a favourable prognosticator in patients with pancreatic cancer. *British Journal of Cancer*.

[91] Dieu-Nosjean M., Giraldo N. A., Kaplon H., Germain C., Fridman W. H., Sautès-Fridman C. (2016). Tertiary lymphoid structures, drivers of the anti-tumor responses in human cancers. *Immunological Reviews*.

[92] Kroeger D. R., Milne K., Nelson B. H. (2016). Tumor-infiltrating plasma cells are associated with tertiary lymphoid structures, cytolytic T-cell responses, and superior prognosis in ovarian cancer. *Clinical Cancer Research*.

[93] Goc J., Germain C., Vo-Bourgais T. K. D. (2014). Dendritic cells in tumor-associated tertiary lymphoid structures signal a th1 cytotoxic immune contexture and license the positive prognostic value of infiltrating CD8+ t cells. *Cancer Research*.

[94] Joshi N. S., Akama-Garren E. H., Lu Y. (2015). Regulatory T cells in tumor-associated tertiary lymphoid structures suppress anti-tumor T cell responses. *Immunity*.

[95] Gobert M., Treilleux I., Bendriss-Vermare N. (2009). Regulatory T cells recruited through CCL22/CCR4 are selectively activated in lymphoid infiltrates surrounding primary breast tumors and lead to an adverse clinical outcome. *Cancer Research*.

[96] Finkin S., Yuan D., Stein I. (2015). Ectopic lymphoid structures function as microniches for tumor progenitor cells in hepatocellular carcinoma. *Nature Immunology*.

[97] Noort A. R., van Zoest K. P. M., van Baarsen L. G. (2015). Tertiary lymphoid structures in rheumatoid arthritis: NF-*κ*B-inducing kinase-positive endothelial cells as central players. *The American Journal of Pathology*.

[98] Carrega P., Loiacono F., Di Carlo E. (2015). NCR + ILC3 concentrate in human lung cancer and associate with intratumoral lymphoid structures. *Nature Communications*.

[99] Withers D. R., Hepworth M. R., Wang X. (2016). Transient inhibition of ROR-*γ*t therapeutically limits intestinal inflammation by reducing TH17 cells and preserving group 3 innate lymphoid cells. *Nature Medicine*.

[100] Sawa S., Lochner M., Satoh-Takayama N. (2011). ROR*γ*t^+^ innate lymphoid cells regulate intestinal homeostasis by integrating negative signals from the symbiotic microbiota. *Nature Immunology*.

[101] Killig M., Glatzer T., Romagnani C. (2014). Recognition strategies of group 3 innate lymphoid cells. *Frontiers in Immunology*.

[102] Deerinck T. J. (2008). The application of fluorescent quantum dots to confocal, multiphoton, and electron microscopic imaging. *Toxicologic Pathology*.

[103] Eissing N., Heger L., Baranska A. (2014). Easy performance of 6-color confocal immunofluorescence with 4-laser line microscopes. *Immunology Letters*.

[104] Wlodarczyk J., Woehler A., Kobe F., Ponimaskin E., Zeug A., Neher E. (2008). Analysis of FRET signals in the presence of free donors and acceptors. *Biophysical Journal*.

[105] Conchello J.-A., Lichtman J. W. (2005). Optical sectioning microscopy. *Nature Methods*.

[106] Sumen C., Mempel T. R., Mazo I. B., von Andrian U. H. (2004). Intravital microscopy: visualizing immunity in context. *Immunity*.

[107] Bajénoff M., Egen J. G., Koo L. Y. (2006). Stromal cell networks regulate lymphocyte entry, migration, and territoriality in lymph nodes. *Immunity*.

[108] Gibson V. B., Benson R. A., Bryson K. J. (2012). A novel method to allow noninvasive, longitudinal imaging of the murine immune system in vivo. *Blood*.

[109] Lappin M. B., Weiss J. M., Delattre V. (1999). Analysis of mouse dendritic cell migration in vivo upon subcutaneous and intravenous injection. *Immunology*.

[110] Sellers S. L., Payne G. W. (2011). Intravital microscopy of the inguinal lymph node. *Journal of Visualized Experiments*.

[111] Irshad S., Flores-Borja F., Evans R. (2013). Use of live in-vivo lymphatic imaging techniques to study the effects of immune cell interactions in a syngeneic mouse model of breast cancer. *Annals of Oncology*.

[112] Ito K., Smith B. R., Parashurama N. (2012). Unexpected dissemination patterns in lymphoma progression revealed by serial imaging within a murine lymph node. *Cancer Research*.

[113] Schafer R., Leung H. M., Gmitro A. F. (2014). Multi-modality imaging of a murine mammary window chamber for breast cancer research. *BioTechniques*.

[114] Kedrin D., Gligorijevic B., Wyckoff J. (2008). Intravital imaging of metastatic behavior through a mammary imaging window. *Nature Methods*.

[115] Looney M. R., Thornton E. E., Sen D., Lamm W. J., Glenny R. W., Krummel M. F. (2011). Stabilized imaging of immune surveillance in the mouse lung. *Nature Methods*.

[116] De Grove K. C., Provoost S., Verhamme F. M. (2016). Characterization and quantification of innate lymphoid cell subsets in human lung. *PLoS ONE*.

[117] Tomura M., Yoshida N., Tanaka J. (2008). Monitoring cellular movement in vivo with photoconvertible fluorescence protein ‘Kaede’ transgenic mice. *Proceedings of the National Academy of Sciences of the United States of America*.

